# Unveiling Microbiota Profiles in Saliva and Pancreatic Tissues of Patients with Pancreatic Cancer

**DOI:** 10.3390/microorganisms13010119

**Published:** 2025-01-09

**Authors:** Alper Uguz, Can Muftuoglu, Ufuk Mert, Tufan Gumus, Deniz Ece, Milad Asadi, Ozlem Ulusan Bagci, Ayse Caner

**Affiliations:** 1 Department of General Surgery, Faculty of Medicine, Ege University, 35040 Izmir, Turkey; alper.uguz@ege.edu.tr (A.U.); tufan.gumus@ege.edu.tr (T.G.); 2 Department of Basic Oncology, Institute of Health Sciences, Ege University, 35100 Izmir, Turkey; canmuftuoglu1990@gmail.com (C.M.); denizece6716@gmail.com (D.E.); miladasadi1389@gmail.com (M.A.); oubagci@ankara.edu.tr (O.U.B.); 3 Department of Medicine I, University Medical Centre Hamburg-Eppendorf, Martinistr. 52, 20246 Hamburg, Germany; 4 Atatürk Vocational School of Health Services, Ege University, 35100 Izmir, Turkey; ufuk.mert@ege.edu.tr; 5 Translational Pulmonary Research Center (EGESAM), Ege University, 35100 Izmir, Turkey; 6 Department of Microbiology, Faculty of Medicine, Ankara University, 06230 Ankara, Turkey; 7 Department of Parasitology, Faculty of Medicine, Ege University, 35100 Izmir, Turkey

**Keywords:** pancreatic ductal adenocarcinoma, distal cholangiocarcinoma, ampullary cancer, oral microbiome, intrapancreatic microbiome, saline control samples

## Abstract

The pancreas, previously considered a sterile organ, has recently been shown to harbor its own microbiota that may influence tumor biology and patient outcomes. Despite increasing interest in the impact of the microbiome on cancer, the relationship between pancreatic tissue and oral microbiomes in pancreatic ductal adenocarcinoma (PDAC) remains limited. In this study, the oral and pancreas tissue microbiomes of patients with PDAC were compared to patients with other periampullary cancers (DC/AC) and a healthy control group using 16S rRNA gene sequence analysis. The results showed a significant reduction in microbial diversity in the saliva of cancer patients compared to healthy controls, while the PDAC patients exhibited a distinct microbial profile in their pancreatic tissues, consisting predominantly of Firmicutes, Proteobacteria, and Actinobacter, after filtering the microbiome of the indoor environment. Notably, the presence of oral bacteria such as *Anoxybacillus*, *Clostridium*, and *Bacillus* in pancreatic tissues suggests potential translocation from the oral cavity. This study emphasizes the importance of understanding the role of body fluid and tissue microbiota in pancreatic cancer, proposing that oral dysbiosis may contribute to disease progression. Moreover, the results suggest that the microbiome of the indoor environment in which samples are collected and analyzed is also important in microbiota analysis studies.

## 1. Introduction

Pancreatic ductal adenocarcinoma (PDAC) remains one of the most lethal malignancies, characterized by late-stage diagnosis, rapid progression, and limited therapeutic options. Despite advancements in understanding of its molecular and genetic landscape, the survival rate of pancreatic cancer patients is still very low [1,2]. Recently, the human microbiota, the vast consortium of microorganisms residing in and on the human body, has now been recognized as a critical player in maintaining homeostasis and influencing cancer. The role of microbiota in cancer development, progression, and treatment response has garnered significant attention [3,4].

Historically, the pancreas was considered a sterile organ due to its harsh enzymatic environment. However, emerging evidence has challenged this notion, revealing the presence of a distinct microbiota within the pancreatic tissue and its association with pancreatic cancer. Studies have shown that the pancreatic microbiota can influence tumor biology, modulate the immune microenvironment, and impact patient outcomes [3,5,6]. These findings suggest that the microbiota may not only serve as a biomarker for early detection and prognosis, but also as a potential therapeutic target.

The relationship between the microbiota and PDAC involves complex interactions among bacterial communities, host immune responses, and tumor cells. For instance, certain bacterial species have been implicated in promoting inflammation, a known risk factor for pancreatic carcinogenesis [5,7]. Moreover, the microbiota can influence the efficacy of chemotherapeutic agents and immunotherapies, either through direct metabolic interactions or by modulating the host’s immune response [3,5,6,7]. Understanding these intricate relationships is crucial for developing microbiota-based interventions and improving therapeutic strategies for pancreatic cancer.

Despite promising insights, the study of pancreatic microbiota is still in its infancy, with many unanswered questions regarding the composition, functional roles, and mechanisms by which these microbial communities influence pancreatic cancer. This study aims to provide an overview of the current knowledge on the microbiota of oral and pancreatic tissue and assess the presence of oral bacteria within the pancreatic tissue microbiome, in the context of pancreatic cancer. In this study, we recruited three groups of participants in accordance with the following inclusion criteria: patients with PDAC; patients with periampullary cancer, including distal cholangiocarcinoma (DC) and ampullary cancer (AC); and healthy controls (HCs). We simultaneously identified the oral and pancreas tissue microbiota characteristics of matched PDAC and DC/AC patients, comparing them to the oral microbiota of healthy controls, and investigated the possibility of oral translocation of bacteria into pancreatic tissue.

## 2. Materials and Methods

### 2.1. Study Participants

**Figure 1:**
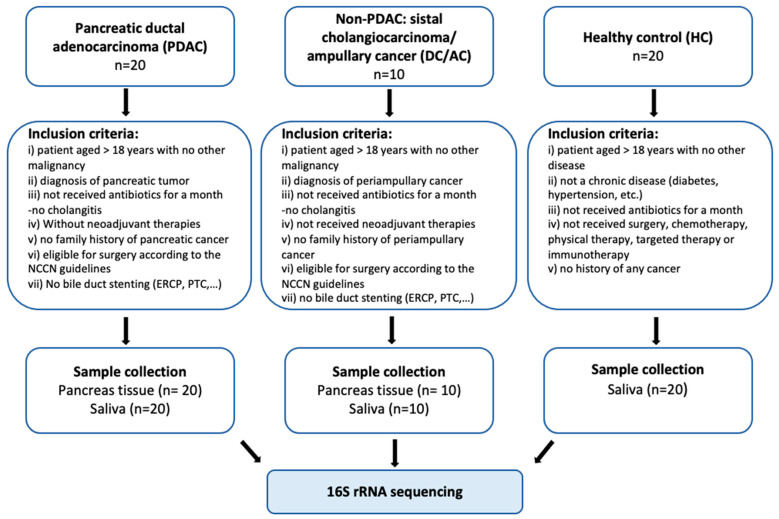
Inclusion criteria of study groups and sampling flow chart.

We enrolled 20 patients with PDAC, 10 patients with DC/AC, and 20 healthy individuals between April 2021 and December 2023. All patients had newly diagnosed and confirmed PDAC or DC/AC and were operable with a pancreaticoduodenectomy (Whipple procedure) [8]. A pancreaticoduodenectomy with regional lymph node dissection is the standard therapeutic approach for both pancreatic head carcinoma and DC/AC [9]. The population of healthy individuals was matched with cancer patients in terms of age, gender, and body mass index (BMI). All participants were aged 33 or over. To exclude conditions affecting the commensal microbial composition, patients who had not received antibiotics/probiotics/proton pump inhibitors/anticancer treatment in the past 8 weeks and who did not have any infectious diseases were included in the study. The inclusion criteria of all the participants and the study design are shown in Figure 1. Patient-matched saliva and tissue samples, including non-pancreatic cancer tissue samples, were collected to assess oral/tissue microbiota, and to investigate the possibility of oral translocation of bacteria into pancreatic tissue. To compare the microbiota in the tissue of patients with PDAC, tissue and oral samples from patients with DC/AC who underwent the same surgery were collected. Only saliva samples were collected from the healthy controls.

This study was approved by the Ethics Committee of Ege University, with the reference number 21-4.1T/48, on 24 February 2024. All the methods were performed in accordance with the relevant guidelines and regulations outlined by the Declaration of Helsinki and the special review board. Informed consent was obtained from all participants prior their participation in the study.

### 2.2. Collection of Samples

Sample collection, processing and storage were conducted with the uniform protocols. Participants who were to provide saliva samples were required to have not eaten anything for at least 8 h and to have ceased smoked for at least 1 h before sampling. Participants also performed their latest dental care routine (brushing teeth, gargling, flossing) the night before giving a saliva sample. The samples were taken into sterile containers in a special sampling room between 07:00 and 09:00 in the morning. Pancreas tissue samples were taken directly in the operating room under sterile conditions by a pancreaticoduodenectomy. With this procedure, after the tumor-containing pancreatic head was removed, the tissue samples were obtained from the body of the pancreas, just around the duct at the surgical site. All samples were immediately aliquoted and stored at −80 °C until further processing. A total of 30 saliva/tissue sample pairs (from 20 PDAC and 10 DC/AC patients) and 20 saliva samples (from the HC group) were finally enrolled. In addition, three saline samples each were taken from the sample collection room (SCR) and the operating room (OR) on three different days. They were subjected to the same processes as the other examples.

### 2.3. DNA Extraction

All DNA extractions were performed in a class II biosafety cabinet. Before the extraction procedure, the tissue samples (20–30 mg) were cut into small pieces with sterile blades. Mechanical disruption of the samples was performed using 0.5 mm glass beads in a MagNA Lyser (Roche, Basel, Switzerland) at 5000 xrpm for 1 min. Then, DNA was extracted from the saliva and tissue samples using a DNeasy blood and tissue kit (Qiagen, Hilden, Germany), according to manufacturer’s instructions. The quality and concentration of the DNA were determined using a NanoDrop ND-2000 spectrophotometer (Thermo Scientific Inc., Waltham, MA, USA) and a Qubit fluorescence assay (Life Technologies, Carlsbad, CA, USA). The isolated DNA was stored at −80 °C until sequencing analysis.

### 2.4. 16S rRNA Sequencing

The V3-V4 hypervariable regions of the bacterial 16S rRNA gene were amplified by the universal primers 16S-forward (CCTACGGGNGGCWGCAG) and 16S-reverse (GACTACHVGGGTATCTAATCC) [10]. A two-step PCR (polimerase chain reaction) process was implemented during library preparation. The PrimeSTAR GXL DNA Polymerase enzyme (Takara, Kusatsu, Japan) was used to conduct a 25-cycle PCR for each sample. The conditions for the first PCR step were as follows: initial denaturation at 95 °C for 3 min, followed by 25 cycles of denaturation at 95 °C for 30 s, annealing at 55 °C for 30 s, and extension at 72 °C for 30 s, with a final extension step at 72 °C for 5 min. In the second PCR step, Illumina index and adapter sequences were added using the Nextera XT Index Primer 1 and Nextera XT Index Primer 2 sets (Illumina, San Diego, CA, USA). The conditions for this PCR were as follows: initial denaturation at 95 °C for 3 min, followed by 8 cycles of denaturation at 95 °C for 30 s, annealing at 55 °C for 30 s, and extension at 72 °C for 30 s, with a final extension step at 72 °C for 5 min. Following both PCR steps, purification of the PCR products was performed using Agencourt AMPure XP (Beckman Coulter, Brea, CA, USA). The concentration of the prepared libraries was measured using a Qubit fluorometer (Life Technologies, Carlsbad, CA, USA). The sequencing was performed using the NovaSeq 6000 next-generation sequencing platform (Illumina) and the NovaSeq 6000 S4 Reagent Kit (Illumina) with paired-end (2 × 150 bp) reads, following the manufacturer’s guidelines.

### 2.5. Bioinformatic Data Processing

Following the sequencing process, quality control of the obtained read data was performed using the FASTQC tool (v 0.12.0). Based on the quality control results, the data quantity, read quality, GC distribution, k-mer distribution, and potential adapter contamination for each sample were examined. During the sequencing process, low-quality base reads and potential adapter-index contaminations in the raw read data were trimmed using the Trimmomatic (v0.39) tool with quality scores to prevent deviations in the subsequent analysis steps [11]. For taxonomic profiling, reads were aligned to the target organisms using the Kraken2 tool with the RefSeq (Standard-Full) database [12,13]. After alignment, OTU groups in each sample were determined. The R vegan package was used to calculate diversity indices [14]. R scripts and GraphPad Prism were employed for data reporting, statistical analyses, and data visualization.

### 2.6. Statistical Analysis

All statistical analyses were performed with the GraphPad Prism software version 9 (San Diego, CA, USA) and R program (www.r-project.org, R 4.3.1). The differences among the two and three groups were compared with Student’s *t* and ANOVA tests, respectively. Continuous and categorical variables were compared between the groups using the Wilcoxon test, Mann–Whitney U test, Kruskal–Wallis test, and Fisher’s exact test. A *p* value of <0.05 was taken as significant.

## 3. Results

### 3.1. Characteristics of Participants

In this study, we included a total of 50 individuals, of which 20, 10, and 20 were PDAC patients, DC/AC patients, and HC individuals, respectively. A total of 50 saliva and 30 pancreas tissue samples were collected from the participants. The results indicated that the average ages of the PDAC patients, DC/AC patients, and healthy controls were 62.3, 64.2, and 61.9, respectively. There were no significant differences observed in age, BMI, and gender between the PDAC patients, DC/AC patients, and healthy controls (*p* > 0.1) Detailed demographic and clinical features of the enrolled participants are summarized in Table 1.

### 3.2. Bacterial Diversity in Pancreas Tissue and Saliva of Cancer Patients

**Figure 2:**
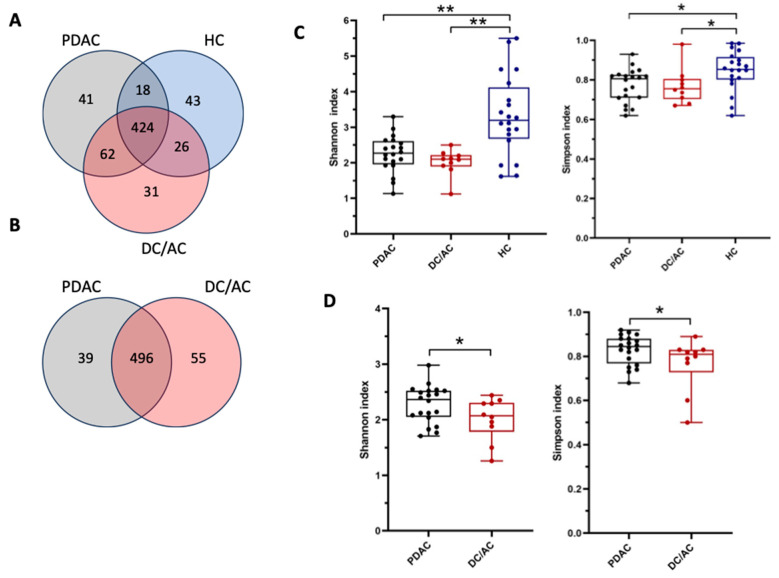
Coverage of taxonomies in saliva and pancreatic tissue samples. Venn diagrams illustrate overlap of OTUs in microbiota of saliva (**A**) and pancreatic tissue (**B**) samples. Red circle means periampullary cancer including distal cholangiocarcinoma/ampullary cancer (DC/AC), gray circle means pancreatic ductal adenocarcinoma (PDAC), and blue circle means healthy control (HC). Alpha diversity shows differences in saliva samples (**C**) and tissue samples (**D**) of PDAC, DCC, and HC groups. * *p* < 0.05; ** *p* < 0.01 indicates significant values in the graph, not significant value isn’t shown in the graph.

In general, an average of 778 operational taxonomic units (OTUs) were identified from 80 samples by 16S rRNA sequence analysis. The OTUs were classified into 19 phyla, 31 classes, 53 orders, 110 families, 178 genera, and 387 species in total. A total of 644 OTUs were detected in saliva samples and 590 OTUs in tissue samples. The following Venn diagrams show overlapping and divergent OTUs between the groups (Figure 2A,B).

The diversity differed between the groups. In the saliva samples, the Shannon and Simpson indices were significantly different between the HC and cancer patient samples (*p* < 0.01 and *p* < 0.05, respectively). However, no significant differences were found between the samples of the PDAC and DC/AC patients. The salivary microbial diversity of the cancer patients was markedly reduced compared to the HCs (Figure 2C). On the other hand, both indices showed that the tissue samples of the PDAC patients exhibited significantly more diversity compared to those of the DC/AC patients (*p* < 0.05) (Figure 2D).

### 3.3. Taxonomic Comparison of Microbial Communities in Saliva and Tissue Samples

**Figure 3:**
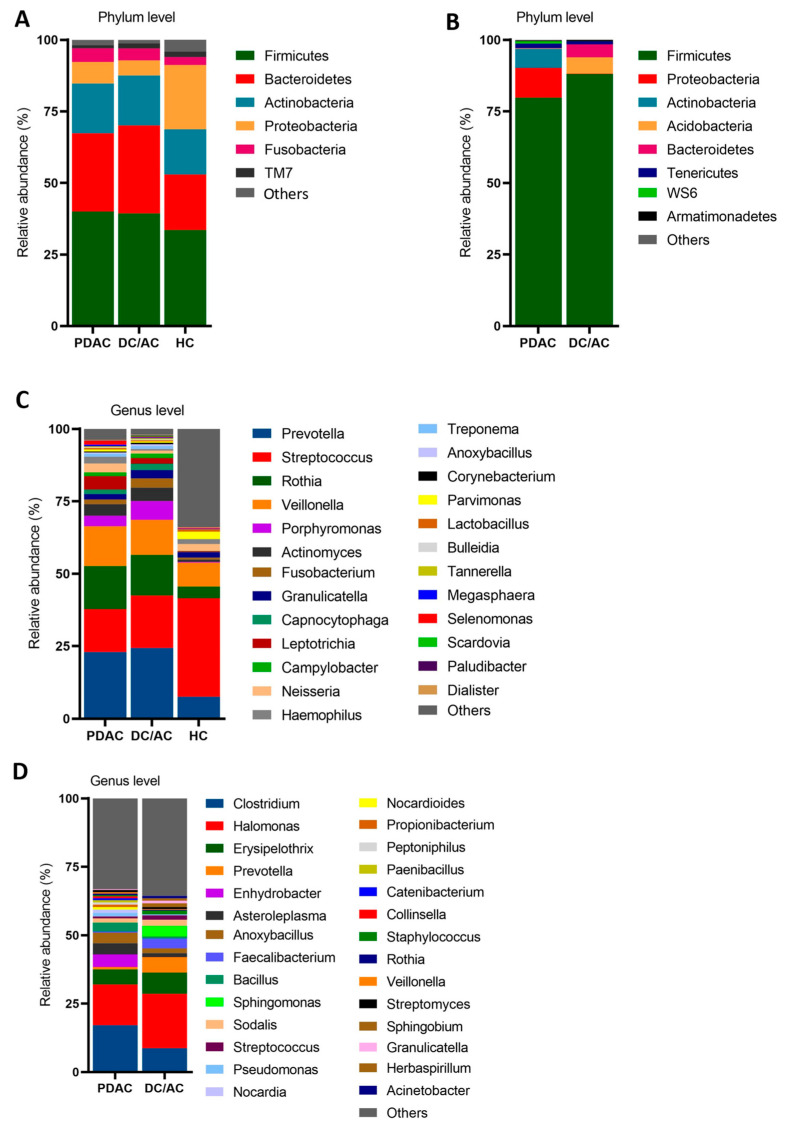
The microbiome profiles in the samples. The relative abundances of each phylum in the saliva samples (**A**) and pancreatic tissue samples (**B**) are shown. (**A**) The relative abundances of each genus in the saliva samples (**C**) and pancreatic tissue samples (**D**) are shown.

A taxonomic comparison of the microbial communities in patients with PDAC revealed a different bacterial composition in the tissue and saliva samples compared to those in the healthy control and DC/AC patient groups. Differential abundance analysis was performed after filtering the datasets of control samples consisting of saline. The microbial compositions were discussed at the levels of phylum, genus, and species among the groups. In the saliva samples, Firmicutes, Bacteroidetes, Actinobacteria, Proteobacteria, and Fusobacteria were the most dominant phyla in the groups, accounting for more than 90% of the relative abundance. Firmicutes was the most abundant phylum of all the groups. Overall, a similar phylum abundance was observed between the PDAC and DC/AC patients, but there was a marked difference compared to the HC group. The abundances of Firmicutes and Bacteroides were increased in the cancer patients, while Proteobacteria were significantly enriched in the HC group (Figure 3A).

Moreover, Firmicutes, Actinobacteria, Proteobacteria, Tenericutes, Bacteroidetes, and Acidobacteria were the most dominant phyla in the pancreatic tissue samples of the PDAC and DC/AC patients, accounting for more than 95% of the relative abundance. Similarly to the saliva samples, Firmicutes was the most abundant phylum for both groups. However, the abundances of Proteobacteria and Actinobacteria were significantly increased in the PDAC group, while Bacteroidetes and Acidobacteria were enriched in the DC/AC patients (Figure 3B).

At the genus level, the microbiota composition changed significantly in the oral samples. Specially, *Streptococcus*, *Provetella*, *Rothia*, *Veillonella*, *Acytinomyces*, *Porphyromonas*, and *Fusobacterium* were the dominant bacteria in the oral samples. Among these, *Provetella*, *Rothia*, *Veillonella*, *Porphyromonas*, *Acytinomyces*, and *Fusobacterium* exhibited significant enrichment in patients with PDAC and DC/AC, while *Streptococcus* was more abundant in the HC group. Although an overall similar microbiota was observed between the PDAC and DC/AC patients, *Streptococcus*, *Porphyromonas*, *Acytinomyces*, and *Fusobacterium* were more abundant in the DC/AC patients than in the PDAC patients, and *Rothia* and *Leptotrichia* were enriched in the PDAC group (Figure 3C).

The bacterial genera found in the pancreatic tissues were different compared to those found in the saliva, and varied greatly among patients. In the tissues, the abundances of *Clostridium*, *Halomonas*, *Bacillus*, *Erysipelothrix*, *Enhydrobacter*, *Sphingomonas*, *Anoxybacillus*, *Prevotella*, and *Asteroleplasma* were significantly enriched. While *Clostridium*, *Enhydrobacter*, *Anoxybacillus*, *Bacillus*, and *Asteroleplasma* were abundant in the PDAC patients, *Halomonas*, *Erysipelothrix*, *Prevotella*, and *Sphingomonas* were significantly enriched in the DC/AC patients (Figure 3D).

**Figure 4:**
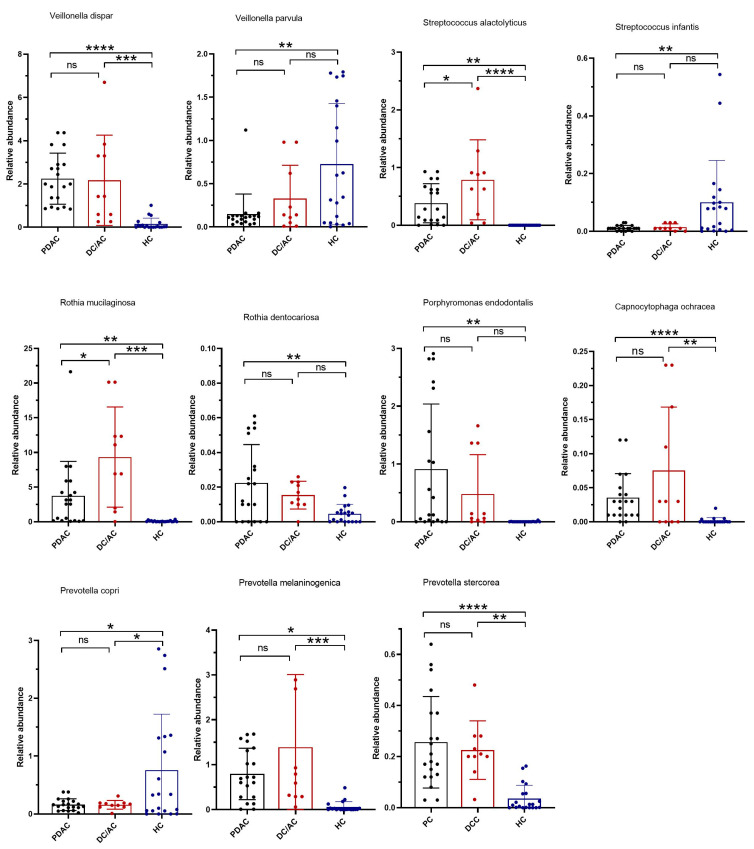
Relative abundance of significantly enriched species in saliva samples of the three groups: pancreatic ductal adenocarcinoma (PDAC) (gray circle), distal cholangiocarcinoma/ampullary cancer (DC/AC) (red circle), and healthy control (HC) (blue circle). *, **, ***, and **** represent *p* < 0.05, *p* < 0.01, *p* < 0.001, and *p* < 0.0001, respectively.

**Figure 5:**
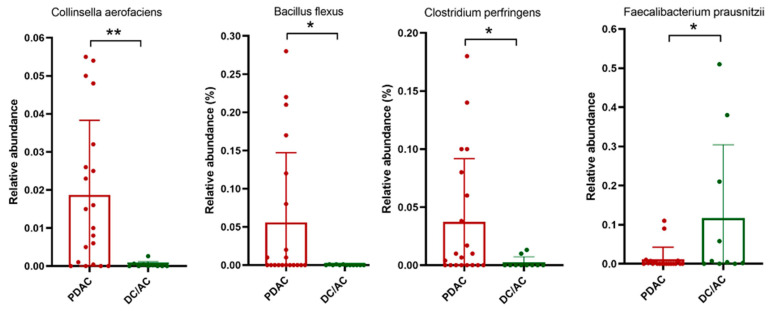
Relative abundance of significantly enriched species in pancreatic tissue samples of the cancer patients: pancreatic ductal adenocarcinoma (PDAC) (red circle) and distal cholangiocarcinoma/ampullary cancer (DC/AC) (green circle). * and ** represent *p* < 0.05 and *p* < 0.01, respectively.

At the species level, taxa and relative abundance were found to vary markedly among patients and sample species. The most common species were *Veillonella dispar*, *Rothia mucilaginosa*, *Rothia dentocariosa*, *Porphyromonas endodontalis*, *Provetella melaninogenica*, *Provetella stercorea*, *Streptococcus alactolyticus*, and *Capnocytophaga ochracea*, which were present in 30% of the saliva samples of the patient groups (Figure 4). In terms of the tissue microbiome, the abundances of *Colinsella aerofaciens*, *Bacillus flexus*, and *Clostridium perfiringes* were found to be significantly increased in the PDAC patients compared to the DC/AC patients, while *Faecalibaterium prausnitzii* revealed a marked enrichment in patients with DC/AC (Figure 5).

### 3.4. Microbial Communities in Matched Saliva and Tissue Samples

**Figure 6:**
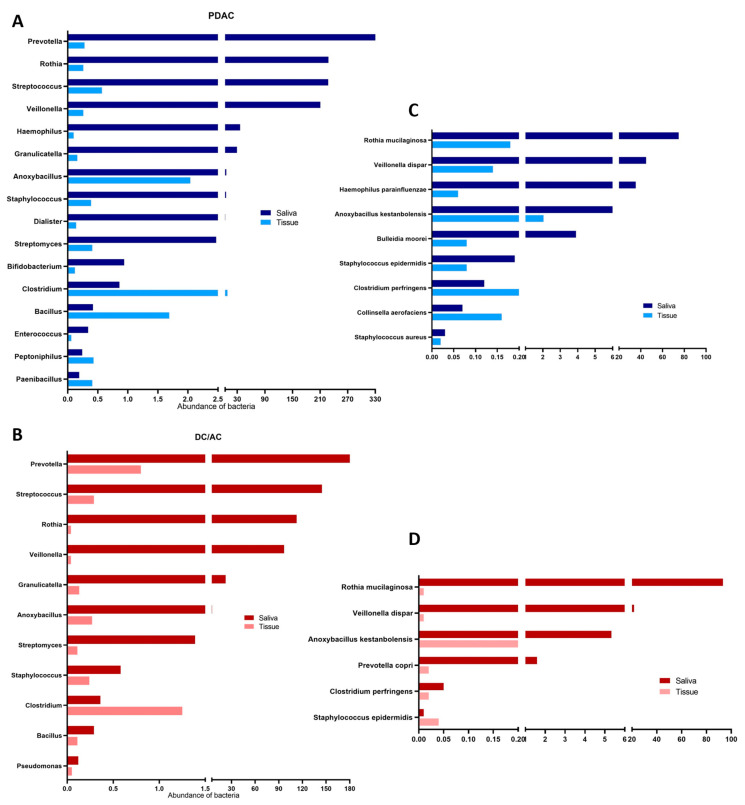
The microbiome profiles in the saliva and pancreatic tissue samples of matched cancer patients. The distributions of significant bacterial abundance at the genus (**A**,**B**) and species (**C**,**D**) levels are shown.

To investigate the origin of the tissue microbiome in cancer patients, the sequences of the pancreatic tissue and saliva samples of matched patients were compared. The findings of this study revealed the relationship between the oral microbiota and pancreatic microbiome in PDAC and DC/AC patients. The genera found in the saliva were present in at least 20–25% of the pancreatic tissues of both patient groups. Notably, *Streptococcus*, *Anoxybacillus*, *Clostridium*, and *Bacillus* were the most prevalent and abundant taxa, found in nearly all of the matched sample pairs of the PDAC patients (Figure 6A). On the other hand, the prevalence of taxa that were commonly detected in the matched sample pairs of the DC/AC patients were much lower than in the PDAC patients, with *Prevotella*, *Streptococcus*, and *Clostridium* being the most abundant taxa in common between the groups (Figure 6B).

At the species level, taxa and relative abundance varied more significantly between the patient samples. The most common species were *Rothia mucilaginossa* and *Veillonella dispar* in the oral and tissue samples of the matched PDAC patients (Figure 6C). Among the species detected in the saliva samples, *Anoxybacillus kestanbolensis* was the most abundant species in the tissue samples of both the PDAC and DC/AC patients (Figure 6D). Interestingly, the pancreatic tissues of the PDAC patients showed a very high abundance of *Clostridium perfringens*, compared to the saliva samples of the PDAC patients and both types of samples of the DC/AC patients (Figure 6C,D).

**Figure 7:**
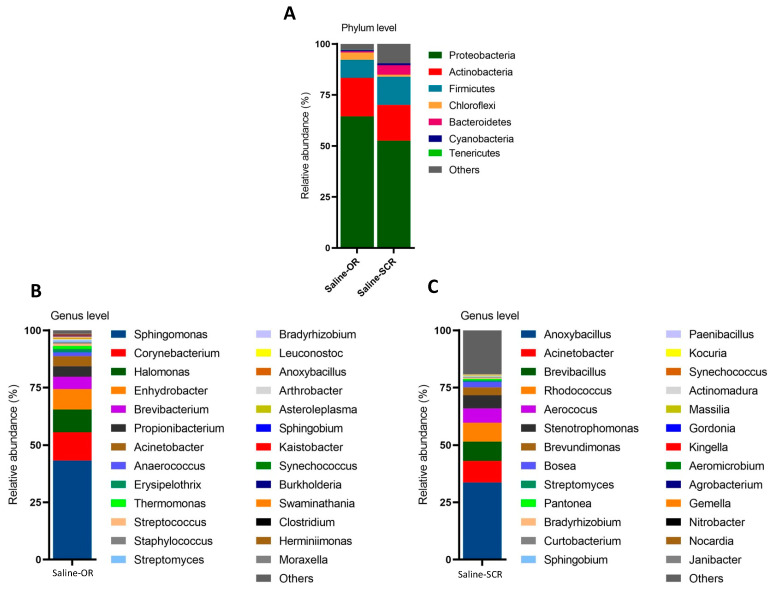
Relative abundance of top bacteria at phylum (**A**) and genus (**B**,**C**) levels, according to indoor environments in each sample collection room. Saline—OR (operating room), Saline—SCR (sample collection room).

**Figure 8:**
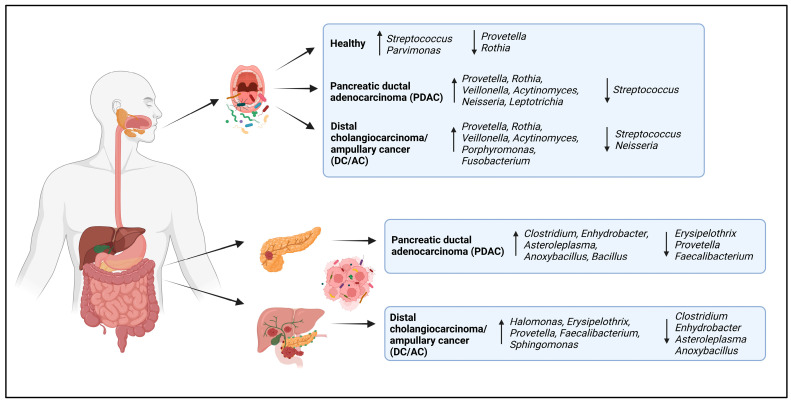
The summary of oral and pancreatic tissue microbiomes in PDAC, DC/AC, and HC groups. The direction of the arrows in the boxes shows the increase and decrease in bacterial abundance.

Moreover, to determine environmental contamination, 16S rRNA analysis was also performed on saline samples taken from the sampling collection room (SCR) and operating room (OR) on three different days. These samples, obtained under similar conditions, were subjected to the same analysis process, as a result of which some microbial communities were identified. In general, the richness and abundance of the microbial compositions were very low in these samples, although a few bacterial communities were remarkably enriched. According to the relative abundance of phylum detected only in saline, *Proteobacteria*, *Actinobacteria*, *Firmicutes*, *Chloroflexi*, and *Bacteroidetes* were the dominant bacteria (Figure 7A). At the genus level, *Anoxybacillus*, *Acinetobacter*, *Brevibacillus*, *Rhodococcus*, *Aerococcus*, and *Stenotrophomonas* were the most abundant bacterial genera in only the saline samples from SCR (Figure 7B), while *Sphingomonas*, *Corynebacterium*, *Halomonas*, and *Enhydrobacter* were the predominant genera in the salina samples from OR (Figure 7C). The oral and tissue microbiomes in the PDAC, DC/AC, and HC groups are summarized in Figure 8.

## 4. Discussion

In this study, we comprehensively characterized the oral and pancreatic tissue microbiota of PDAC and DC/AC patients using 16S rRNA analysis. Since we could not obtain pancreatic tissue samples from healthy individuals, we compared the tissue microbiota from PDAC patients with those from DC/AC patients. PDAC and DC/AC are characterized by anatomical proximity, morphological similarity, and an overlapping immunohistochemical profile. PDAC and DC/AC are very aggressive neoplasms, and effective treatments are still limited [15]. With the pancreaticoduodenectomy procedure, which involves the removal of the head of the pancreas [8], we obtained pancreatic tissues for both diseases. However, the oral microbiota of PDAC patients were compared to that of both DC/AC patients and an HC group.

Understanding the content of the oral microbiome in health and disease conditions makes it a promising approach for cancer diagnosis. Previous studies have reported that an imbalance in oral microbiota composition is associated with pancreatic cancer pathogenesis, and some imbalances in composition may be potential diagnostic biomarkers [16,17,18]. Similarly to studies showing oral microbial diversity in PDAC patients [19,20,21], the present study showed that the microbial diversity of saliva samples was remarkably lower in PDAC and DC/AC patients compared to the HC group, while no significant difference was observed between the PDAC and DC/AC groups. The findings exhibited striking changes in the oral microbial composition of cancer patients compared to the HC group, suggesting that an increase in the richness or diversity of the microbiota is an indicator of health [22,23]. On the other hand, low microbial diversity in patients with PDAC and DC/AC may indicate an overgrowth of various harmful or pathogenic bacteria related to these diseases [20].

Nowadays, the presence of bacteria in pancreatic tissues, previously thought to be a sterile organ, is well known. Similarities in the human duodenum and pancreatic microbiomes suggest that bacteria may directly translocate from the duodenum to the pancreas [24]. Recent studies have confirmed the presence of bacteria within the pancreas in PDAC and its potential influence on tumor growth [25]. Moreover, studies have reported that PDAC in patients with more bacterial diversity showed enhanced immune infiltration and favorable prognosis compared to PDAC in patients with less bacterial diversity [26]. This study revealed that the tissue microbial diversity of the PDAC group was significantly higher than that of the DC/AC group. Although PDAC and DC/AC are generally associated with poor survival, in contrast to the microbial findings in this study, patients with DC/AC seem to exhibit a better prognosis [15]. However, while microbiota studies have been performed on the intestinal gut and bile ducts of DC/AC patients, there is no information on the microbiota of the pancreatic tissue of these patients.

We have shown that the bacteria in the pancreatic tissue of PDAC patients exhibited a different profile than in the tissue of DC/AC patients, and were predominantly composed of Firmicutes, Proteobacteria, and Actinobacter phyla, which is consistent with previous reports [25,27]. However, Bacteroidetes and Acidobacteria were the most abundant phyla in the DC/AC patients, and Firmicutes colonized more than in the tissues of PDAC patients. Although it is controversial whether the microbial profile within tumoral pancreatic tissues in PDAC patients differs from that in non-tumoral pancreatic tissues [24,28], comparative analyses have shown that the colonization of different bacterial species within PDAC differs from those in the surrounding tissue. Particularly, the highest abundance of bacteria in human PDAC at the phylum level was Proteobacteria, reaching approximately 30–70% [25,27,29,30]. However, we detected the highest abundance of Proteobacteria in saline samples from the operating room. After filtering the serum saline microbiota, Firmicutes were present in more than 75% of the tissue samples in this study. Similarly, Fu et al. showed that Proteobacteria accounted for 56.2% of all bacteria in the indoor air microbiome, followed by Firmicutes (21.5%), Actinobacteria (9.0%), Deinococcus-Thermus (6.0%), Fusobacteriia (3.9%), and Bacteroidetes (2.0%) [31]. In this case, the findings suggest that the microbiome of the indoor air in which the samples are collected and analyzed is as important as the sampling method in microbiota analysis studies.

Oral microbiota composition varied significantly between the cancer patients and healthy controls, and was predominantly composed of phyla consistent with previous reports [20,32,33]. The abundances of Firmicutes, Bacteroidetes, and related taxa were higher in the PDAC and DC/AC patients, while the abundancse of Proteobacteria and related taxa were higher in the HC group [32]. Oral pathogenic genera (*Prevotella*, *Rothia*, *Veillonella*, *Porphyromonas*, *Actinomyces*, *Fusobacterium*, etc.) showed a marked enrichment in patients with PDAC and DC/AC, while *Streptococcus* were the dominant bacteria in the saliva samples of the HC group. The composition of the oral microbiota in the PDAC patients and healthy subjects showed a similar profile to that reported in other studies [20,32,33,34].

The composition of the oral microbiome of the PDAC and DC/AC patients exhibited a greater diversity than the microbiome of their tissue samples. The abundance of taxa was higher in the pancreatic tissues of the PDAC patients than those of the DC/AC patients. Despite this diversity, oral bacteria, mainly *Prevotella*, *Rothia*, *Streptococcus*, *Veillonella*, *Porphyromonas*, *Actinomyces*, *Corynebacterium*, and *Granulicatella*, remained the dominant bacteria in most of the tissue samples of cancer patients. These bacteria were found in lower abundance within the HC group, whereas they were common in the oral microbiomes of cancer patients, suggesting that oral dysbiosis may be present in patients with PDAC [35]. Studies have also linked poor oral health or periodontal disease to pancreatic cancer, suggesting the possibility that oral bacteria may translocate from the gingiva into the bloodstream and colonize the pancreas tissue of cancer patients [36,37].

An abundance of oral bacteria in PDAC tissues has also been previously described [37,38,39]. In the present study, it was revealed that *Anoxybacillus*, *Clostridium*, and *Bacillus* detected in the saliva samples were the most abundant species in the pancreatic tissue of the PDAC patients, particularly species with *Anoxybacillus kestanbolensis* and *Clostridium perfringens.* The prevalence and abundance of oral bacteria in PDAC tumors may be due to the microenvironment of the tumor, which allows for the recruitment of certain bacteria due to secreted metabolites [3,40,41]. PDAC cells can produce metabolites such as lactate and alanine, and provide hypoxic microenvironments, creating an ideal environment for bacteria to survive and multiply [3,41,42]. In addition, the bacteria here can allow other bacteria to colonize [40].

Moreover, while the field of microbiome studies has become an established area of research and many best practices exist, data on the use of negative controls in studies is scarce. While there are no standards on controls, it is essential that all studies in this area include these controls to avoid erroneous results and improve data interpretation [43,44]. This study provides information supporting the necessity of the microbial analysis of indoor air, which can be used as negative control data in microbiome studies. On the other hand, since there is a potential link between bacterial translocation in oral as well as intestinal dysbiosis and pancreatic cancer, integrating fecal microbiota transplantation into pancreatic cancer treatment could provide significant benefits [45,46].

## 5. Conclusions

There was not much difference between the oral microbiota of PDAC and DC/AC patients, whereas the healthy control group exhibited notable differences. In addition, the tissue microbial communities in patients with PDAC and DC/AC were found to differ significantly, and were enriched with some bacteria from the oral microbiota. The results of this study further support the relationship between the oral microbiome and pancreatic tissue microbiome in patients with PDAC or DC/AC. Further studies are needed to evaluate the impact of oral microbiota on pancreatic tissue and tumor biology in the diagnosis and treatment of PDAC.

## Figures and Tables

**Table 1:** Characteristics of cancer patients and healthy individuals.

Variables	PDAC Patients *n* = 20 (%)	DC/AC Patients *n* = 10 (%)	Healthy Control *n* = 20 (%)	*p*-Value
Age (year, range)	62.3 (34–71)	64.2 (41–67)	61.9 (33–69)	0.346
**Gender**				
Female	6 (30)	2 (20)	7 (35)	0.699
Male	14 (70)	8 (80)	13 (65)	
BMI (kg/m^2^)	29.8 ± 4.4	25.8 ± 6.9	27.5 ± 5.7	0.217
**Smoking status**				
Never	9 (45)	6 (60)	10 (45)	0.362
Former (before 5 years)	6 (30)	0	6 (20)	
Current (20–30 cigarettes/day)	5 (25)	4 (40)	4	
**Alcohol consumption (drinks/day)**				
0–1	11	14	12	0.827
1–4	6	5	6	
More than 4	3	1	2	
**Comorbid diseases**				
Cardiovascular disease	2 (5)	0	2 (5)	0.803
Hypertension	8 (27.5)	2	9 (22.5)	
Diabetes mellitus	2 (10)	1	1 (2.5)	
**Family history of PDAC**	1 (0.5)	0	0	

Pancreatic ductal adenocarcinoma: PDAC; distal cholangiocarcinoma/ampullary cancer: DC/AC; body mass index: BMI.

## Data Availability

The original contributions presented in the study are included in the article, further inquiries can be directed to the corresponding author.

## References

[1] Adamska A., Domenichini A., Falasca M. (2017). Pancreatic Ductal Adenocarcinoma: Current and Evolving Therapies. Int. J. Mol. Sci..

[2] Chen T., Li X., Li G., Liu Y., Huang X., Ma W., Qian C., Guo J., Wang S., Qin Q. (2023). Alterations of commensal microbiota are associated with pancreatic cancer. Int. J. Biol. Markers.

[3] Vilà-Quintana L., Fort E., Pardo L., Albiol-Quer M.T., Ortiz M.R., Capdevila M., Feliu A., Bahí A., Llirós M., García-Velasco A. (2024). Metagenomic Study Reveals Phage-Bacterial Interactome Dynamics in Gut and Oral Microbiota in Pancreatic Diseases. Int. J. Mol. Sci..

[4] Heiman M.L., Greenway F.L. (2016). A healthy gastrointestinal microbiome is dependent on dietary diversity. Mol. Metab..

[5] Hou K., Wu Z.X., Chen X.Y., Wang J.Q., Zhang D., Xiao C., Zhu D., Koya J.B., Wei L., Li J. (2022). Microbiota in health and diseases. Signal Transduct. Target Ther..

[6] Del Castillo E., Meier R., Chung M., Koestler D.C., Chen T., Paster B.J., Charpentier K.P., Kelsey K.T., Izard J., Michaud D.S. (2019). The Microbiomes of Pancreatic and Duodenum Tissue Overlap and Are Highly Subject Specific but Differ between Pancreatic Cancer and Noncancer Subjects. Cancer Epidemiol. Biomark. Prev..

[7] Geller L.T., Barzily-Rokni M., Danino T., Jonas O.H., Shental N., Nejman D., Gavert N., Zwang Y., Cooper Z.A., Shee K. (2017). Potential role of intratumor bacteria in mediating tumor resistance to the chemotherapeutic drug gemcitabine. Science.

[8] Riquelme E., Zhang Y., Zhang L., Montiel M., Zoltan M., Dong W., Quesada P., Sahin I., Chandra V., San Lucas A. (2019). Tumor Microbiome Diversity and Composition Influence Pancreatic Cancer Outcomes. Cell.

[9] Nejman D., Livyatan I., Fuks G., Gavert N., Zwang Y., Geller L.T., Rotter-Maskowitz A., Weiser R., Mallel G., Gigi E. (2020). The human tumor microbiome is composed of tumor type-specific intracellular bacteria. Science.

[10] Pushalkar S., Hundeyin M., Daley D., Zambirinis C.P., Kurz E., Mishra A., Mohan N., Aykut B., Usyk M., Torres L.E. (2018). The Pancreatic Cancer Microbiome Promotes Oncogenesis by Induction of Innate and Adaptive Immune Suppression. Cancer Discov..

[11] Nalluri H., Jensen E., Staley C. (2021). Role of biliary stent and neoadjuvant chemotherapy in the pancreatic tumor microbiome. BMC Microbiol..

[12] Guan S.W., Lin Q., Yu H.B. (2023). Intratumour microbiome of pancreatic cancer. World J. Gastrointest. Oncol..

[13] Fu X., Shama A., Norbäck D., Chen Q., Xia Y., Zhang X., Sun Y. (2024). Exploring the role of indoor microbiome and environmental characteristics in rhinitis symptoms among university students. Front. Microbiomes.

[14] Olson S.H., Satagopan J., Xu Y., Ling L., Leong S., Orlow I., Saldia A., Li P., Nunes P., Madonia V. (2017). The oral microbiota in patients with pancreatic cancer, patients with IPMNs, and controls: A pilot study. Cancer Causes Control.

[15] Okuda S., Hirose Y., Takihara H., Okuda A., Ling Y., Tajima Y., Shimada Y., Ichikawa H., Takizawa K., Sakata J. (2022). Unveiling microbiome profiles in human inner body fluids and tumor tissues with pancreatic or biliary tract cancer. Sci. Rep..

[16] Vogtmann E., Han Y., Caporaso J.G., Bokulich N., Mohamadkhani A., Moayyedkazemi A., Hua X., Kamangar F., Wan Y., Suman S. (2020). Oral microbial community composition is associated with pancreatic cancer: A case-control study in Iran. Cancer Med..

[17] Herremans K.M., Riner A.N., Cameron M.E., McKinley K.L., Triplett E.W., Hughes S.J., Trevino J.G. (2022). The oral microbiome, pancreatic cancer and human diversity in the age of precision medicine. Microbiome.

[18] Maisonneuve P., Amar S., Lowenfels A.B. (2017). Periodontal disease, edentulism, and pancreatic cancer: A meta-analysis. Ann. Oncol..

[19] McKinley K.N.L., Herremans K.M., Riner A.N., Vudatha V., Freudenberger D.C., Hughes S.J., Triplett E.W., Trevino J.G. (2023). Translocation of Oral Microbiota into the Pancreatic Ductal Adenocarcinoma Tumor Microenvironment. Microorganisms.

[20] Galeano Niño J.L., Wu H., LaCourse K.D., Kempchinsky A.G., Baryiames A., Barber B., Futran N., Houlton J., Sather C., Sicinska E. (2022). Effect of the intratumoral microbiota on spatial and cellular heterogeneity in cancer. Nature.

[21] Huang Y., Zhu N., Zheng X., Liu Y., Lu H., Yin X., Hao H., Tan Y., Wang D., Hu H. (2022). Intratumor Microbiome Analysis Identifies Positive Association Between *Megasphaera* and Survival of Chinese Patients With Pancreatic Ductal Adenocarcinomas. Front. Immunol..

[22] Mutha N.V.R., Mohammed W.K., Krasnogor N., Tan G.Y.A., Wee W.Y., Li Y., Choo S.W., Jakubovics N.S. (2019). Transcriptional profiling of coaggregation interactions between Streptococcus gordonii and Veillonella parvula by Dual RNA-Seq. Sci. Rep..

[23] Wu H., Fu M., Wu M., Cao Z., Zhang Q., Liu Z. (2024). Emerging mechanisms and promising approaches in pancreatic cancer metabolism. Cell Death Dis..

[24] Gu I., Gregory E., Atwood C., Lee S.O., Song Y.H. (2022). Exploring the Role of Metabolites in Cancer and the Associated Nerve Crosstalk. Nutrients.

[25] Edmonds K., Williams L. (2018). The Role of the Negative Control in Microbiome Analyses. FASEB J..

[26] Hornung B.V.H., Zwittink R.D., Kuijper E.J. (2019). Issues and current standards of controls in microbiome research. FEMS Microbiol. Ecol..

[27] Brusnic O., Onisor D., Boicean A., Hasegan A., Ichim C., Guzun A., Chicea R., Todor S.B., Vintila B.I., Anderco P. (2024). Fecal Microbiota Transplantation: Insights into Colon Carcinogenesis and Immune Regulation. J. Clin. Med..

[28] Boicean A., Ichim C., Todor S.B., Anderco P., Popa M.L. (2024). The Importance of Microbiota and Fecal Microbiota Transplantation in Pancreatic Disorders. Diagnostics.

[29] Halbrook C.J., Lyssiotis C.A., Pasca di Magliano M., Maitra A. (2023). Pancreatic cancer: Advances and challenges. Cell.

[30] Miyabayashi K., Ijichi H., Fujishiro M. (2022). The Role of the Microbiome in Pancreatic Cancer. Cancers.

[31] Sepich-Poore G.D., Zitvogel L., Straussman R., Hasty J., Wargo J.A., Knight R. (2021). The microbiome and human cancer. Science.

[32] Tijeras-Raballand A., Hilmi M., Astorgues-Xerri L., Nicolle R., Bièche I., Neuzillet C. (2021). Microbiome and pancreatic ductal adenocarcinoma. Clin. Res. Hepatol. Gastroenterol..

[33] Schepis T., De Lucia S.S., Nista E.C., Manilla V., Pignataro G., Ojetti V., Piccioni A., Gasbarrini A., Franceschi F., Candelli M. (2021). Microbiota in Pancreatic Diseases: A Review of the Literature. J. Clin. Med..

[34] Guo X., Wang P., Li Y., Chang Y., Wang X. (2024). Microbiomes in pancreatic cancer can be an accomplice or a weapon. Crit. Rev. Oncol. Hematol..

[35] Karim S.A.M., Abdulla K.S., Abdulkarim Q.H., Rahim F.H. (2018). The outcomes and complications of pancreaticoduodenectomy (Whipple procedure): Cross sectional study. Int. J. Surg..

[36] Hartwig W., Hackert T., Hinz U., Gluth A., Bergmann F., Strobel O., Buchler M.W., Werner J. (2011). Pancreatic cancer surgery in the new millennium: Better prediction of outcome. Ann. Surg..

[37] Klindworth A., Pruesse E., Schweer T., Peplies J., Quast C., Horn M., Glöckner F.O. (2013). Evaluation of general 16S ribosomal RNA gene PCR primers for classical and next-generation sequencing-based diversity studies. Nucleic Acids Res..

[38] Bolger A.M., Lohse M., Usadel B. (2014). Trimmomatic: A flexible trimmer for Illumina sequence data. Bioinformatics.

[39] Wood D.E., Lu J., Langmead B. (2019). Improved metagenomic analysis with Kraken 2. Genome Biol..

[40] Quast C., Pruesse E., Yilmaz P., Gerken J., Schweer T., Yarza P., Peplies J., Glöckner F.O. (2013). The SILVA ribosomal RNA gene database project: Improved data processing and web-based tools. Nucleic Acids Res..

[41] Oksanen J., Blanchet F.G., Kindt R., Legendre P., Minchin P.R., O’hara R.B., Simpson G.L., Solymos P., Stevens M.H.H., Wagner H. (2019). Vegan: Community Ecology Package. R Package Version 2.5-6. https://CRAN.R-project.org/package=vegan.

[42] Gkountakos A., Martelli F.M., Silvestris N., Bevere M., De Bellis M., Alaimo L., Sapuppo E., Masetto F., Mombello A., Simbolo M. (2023). Extrahepatic Distal Cholangiocarcinoma vs. Pancreatic Ductal Adenocarcinoma: Histology and Molecular Profiling for Differential Diagnosis and Treatment. Cancers.

[43] Farrell J.J., Zhang L., Zhou H., Chia D., Elashoff D., Akin D., Paster B.J., Joshipura K., Wong D.T. (2012). Variations of oral microbiota are associated with pancreatic diseases including pancreatic cancer. Gut.

[44] Torres P.J., Fletcher E.M., Gibbons S.M., Bouvet M., Doran K.S., Kelley S.T. (2015). Characterization of the salivary microbiome in patients with pancreatic cancer. PeerJ.

[45] Fan X., Alekseyenko A.V., Wu J., Peters B.A., Jacobs E.J., Gapstur S.M., Purdue M.P., Abnet C.C., Stolzenberg-Solomon R., Miller G. (2018). Human oral microbiome and prospective risk for pancreatic cancer: A population-based nested case-control study. Gut.

[46] Sun H., Zhao X., Zhou Y., Wang J., Ma R., Ren X., Wang H., Zou L. (2020). Characterization of Oral Microbiome and Exploration of Potential Biomarkers in Patients with Pancreatic Cancer. Biomed Res. Int..

